# Severe acute respiratory syndrome coronavirus 2-induced acute aortic occlusion: a case report

**DOI:** 10.1186/s13256-021-02692-x

**Published:** 2021-03-02

**Authors:** Artem Minalyan, Franklin L. Thelmo, Vincent Chan, Stephanie Tzarnas, Faizan Ahmed

**Affiliations:** Department of Medicine, Abington Hospital-Jefferson Health, Abington, PA USA

**Keywords:** COVID-19, Coronavirus, Aortic occlusion, Thrombosis, Case report

## Abstract

**Background:**

Severe acute respiratory syndrome coronavirus 2 infection can lead to a constellation of viral and immune symptoms called coronavirus disease 2019. Emerging literature increasingly supports the premise that severe acute respiratory syndrome coronavirus 2 promotes a prothrombotic milieu. However, to date there have been no reports of acute aortic occlusion, itself a rare phenomenon. We report a case of fatal acute aortic occlusion in a patient with coronavirus disease 2019.

**Case report:**

A 59-year-old Caucasian male with past medical history of peripheral vascular disease presented to the emergency department for evaluation of shortness of breath, fevers, and dry cough. His symptoms started 5–7 days prior to the emergency department visit, and he received antibiotics in the outpatient setting without any effect. He was found to be febrile, tachypneic, and hypoxemic. He was placed on supplemental oxygen via a non-rebreather mask. Chest X-ray showed multifocal opacifications. Intravenous antibiotics for possible pneumonia were initiated. Hydroxychloroquine was initiated to cover possible coronavirus disease 2019 pneumonia. During the hospitalization, the patient became progressively hypoxemic, for which he was placed on bilevel positive airway pressure. D-dimer, ferritin, lactate dehydrogenase, and C-reactive protein were all elevated. Severe acute respiratory syndrome coronavirus 2 reverse transcription polymerase chain reaction was positive. On day 3, the patient was upgraded to the intensive care unit. Soon after he was intubated, he developed a mottled appearance of skin, which extended from his bilateral feet up to the level of the subumbilical plane. Bedside ultrasound revealed an absence of flow from the mid-aorta to both common iliac arteries. The patient was evaluated emergently by vascular surgery. After a discussion with the family, it was decided to proceed with comfort-directed care, and the patient died later that day.

**Discussion:**

Viral infections have been identified as a source of prothrombotic states due to direct injury of vascular tissue and inflammatory cascades. Severe acute respiratory syndrome coronavirus 2 appears to follow a similar pattern, with numerous institutions identifying elevated levels of thrombotic complications. We believe that healthcare providers should be aware of both venous and arterial thrombotic complications associated with coronavirus disease 2019, including possible fatal outcome.

## Background

AAO is a very rare life-threatening condition associated with high mortality despite advances in surgical management, with a 30-day mortality rate of 21–52%. Saddle embolus, *in situ* thrombosis in the setting of severe atherosclerotic disease, and occlusion of previous surgical constructions (for example, stents, grafts) are considered the most common causes of AAO [1]. Abdominal aortic aneurysm (AAA) is another condition that is known to be associated with an increased risk of AAO. It is thought that AAA can cause AAO as there is an interruption in laminar flow leading to turbulence and an increased risk of clot formation [2]. Multiple conditions including malignancies, infections, and connective tissue disorders are known to lead to hypercoagulability. Therefore, patients with said conditions are also at increased risk of thrombotic events [3]. SARS-CoV-2 is the cause of the current 2019–2020 pandemic. There is emerging evidence that it is also associated with hypercoagulable states, therefore, leading to thrombotic events [4]. Arterial thrombotic events have also been described in the literature [5, 6]. It is speculated that endovascular damage triggers the cascade of pathologic changes that leads to the development of arterial thrombi [7].

## Case presentation

A 59-year-old Caucasian male presented to the emergency department (ED) from a nursing home for evaluation of shortness of breath, fevers, and dry cough. His past medical history included schizophrenia, epilepsy, and peripheral vascular disease. As per the patient, his symptoms started 5–7 days prior to the ED visit. He was treated in the outpatient setting with azithromycin for concern of community-acquired pneumonia. He presented to the ED due to progressive dyspnea from his facility where staff found the patient to be hypoxemic with an O_2_ saturation in the 80s on room air. Of note, the patient was exposed to facility residents with confirmed SARS-CoV-2 infection. However, he tested negative for SARS-CoV-2 infection prior to arrival.

**Fig. 1:**
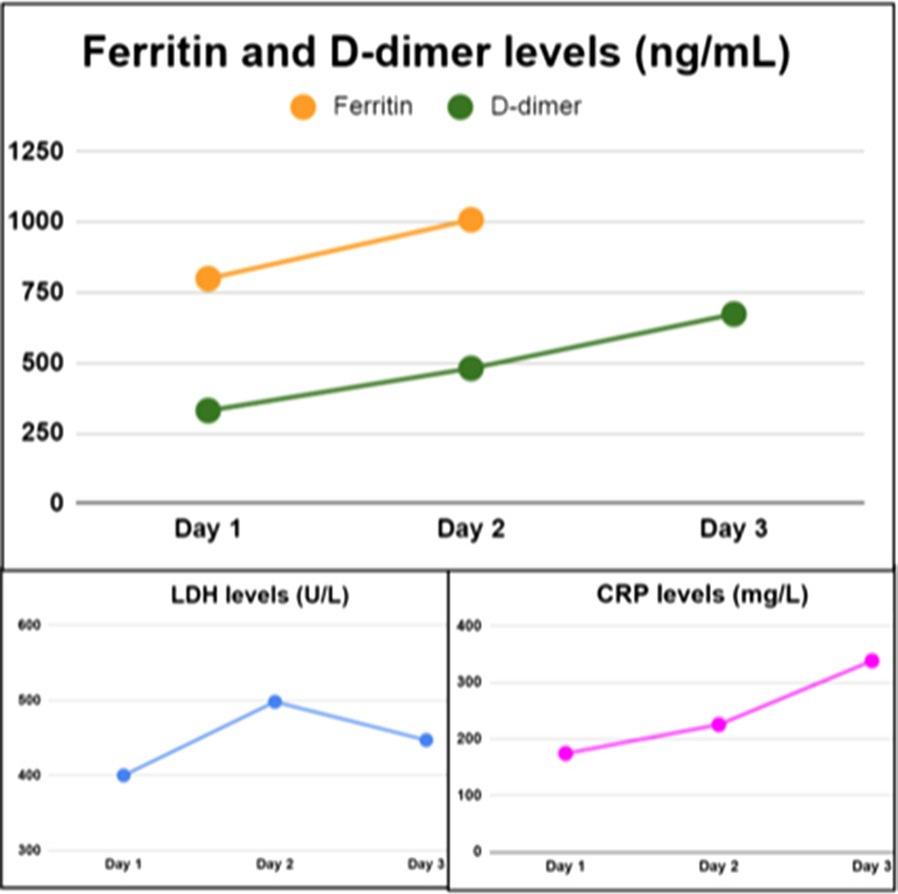
Trends of inflammatory markers during hospitalization

**Fig. 2:**
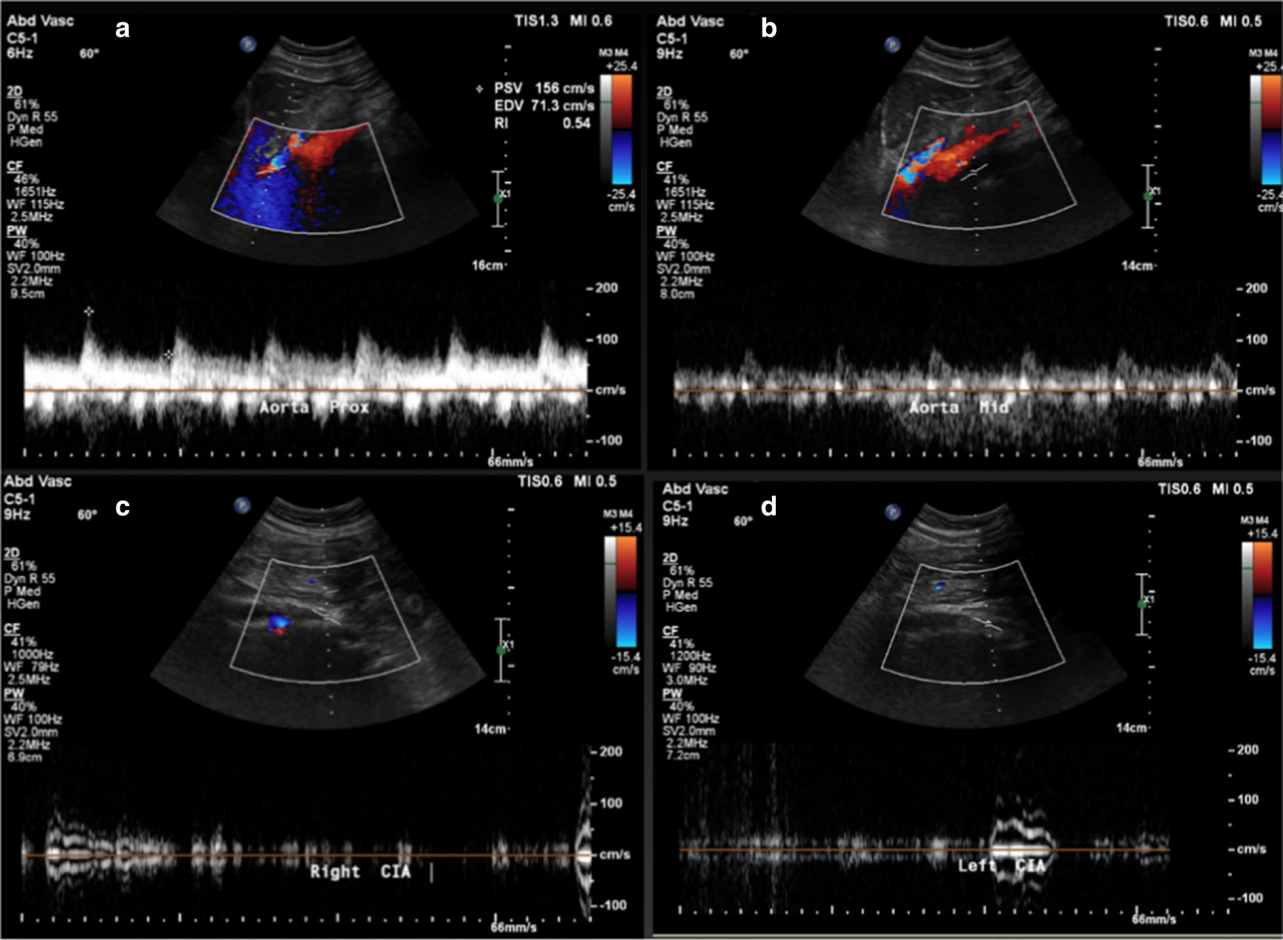
Ultrasound Doppler imaging of patient’s aorta and iliac arteries (**a**) proximal aorta, (**b**) mid aorta, (**c**) left common iliac artery, and (**d**) right common iliac artery

In the ED, he was found to be febrile with oral temperature of 38.2 ℃, tachypnea at 22 breaths per minute, and persistent hypoxemia requiring supplemental oxygen delivery via a non-rebreather mask. Physical exam revealed no aberrations in his heart or lungs. Chest X-ray showed bilateral airway opacities concerning for multifocal pneumonia. A complete metabolic panel (CMP) and complete blood count (CBC) with differential revealed lymphopenia with an absolute lymphocyte count 0.5 K/μL (reference range 1.5–3.2). The patient was started on intravenous antibiotics (vancomycin, cefepime, levofloxacin) for presumed bacterial pneumonia. Hydroxychloroquine was initiated to cover possible COVID-19 pneumonia. The patient was admitted to hospital. On day 2, the patient remained hypoxemic and developed increased work of breathing for which he was placed on bilevel positive airway pressure (BiPAP). D-dimer, ferritin, lactate dehydrogenase, and C-reactive protein were all elevated (Fig. 1). SARS-CoV-2 reverse transcription polymerase chain reaction (RT-PCR) was positive. On day 3, the patient was upgraded to the intensive care unit (ICU) where he was later intubated. Soon after he was intubated, he developed a mottled appearance of skin that extended from his bilateral feet up to the level of the subumbilical plane. Pulses were unable to be detected both via palpation and Doppler. Bedside ultrasound was performed, and absence of anterograde flow was noted from the mid-aorta down to both common iliac arteries (Fig. 2); a deep venous thrombosis (DVT) in the left femoral vein was also revealed. The patient became hypotensive in the ICU with mean arterial pressure (MAP) of 50 mmHg. He was started on a norepinephrine drip for pressure support. Given hemodynamic instability and the high likelihood of venous thromboembolic event, the decision was made to start thrombolytic therapy with tissue plasminogen activator (tPA) of 50 mg intravenously once as well as systemic anticoagulation with a heparin drip. The patient was evaluated emergently by the vascular surgery team. The patient was deemed too unstable to be taken for computed tomography (CT) angiography. No AAA was identified in the patient. After a discussion with the family, it was decided to proceed with comfort-directed care, and the patient died later that day.

## Discussion and conclusions

SARS-CoV-2, first reported in Wuhan, China on 31 December 2019, is a virus that has caused a pandemic as declared by the World Health Organization (WHO) in March 2020 [8, 9]. As of mid-May, there are almost 4,500,000 reported cases worldwide while the USA has the highest number of cases (almost 1,500,000). It is estimated that at least 300,000 people have died so far because of COVID-19 [10]. SARS-CoV-2 belongs to the genus *Betacoronavirus* [11]. There are four lineages within the genus: A (HCoV-OC43, HCoV-HKU1)—frequently found in patients with common cold; B (SARS-CoV and SARS-CoV-2)—cause of SARS and COVID-19 pandemics, respectively; C (Middle East respiratory syndrome-related coronavirus (MERS-CoV)—cause of MERS outbreak; D (CoV-HKU9)—found only in animals [12]. They are all enveloped positive-sense single-stranded ribonucleic acid (RNA) viruses. Notably, both SARS-CoV and SARS-CoV-2 use the same receptor, angiotensin-converting enzyme 2 (ACE2), for cell entry [13]. It is estimated that SARS-CoV-2 is more contagious than seasonal influenza on the basis of their differing *R*_0_ values, with influenza being estimated at 1.3 and SARS-CoV-2 being estimated at 2.5 [14, 15]. The majority of patients with COVID-19 have nonsevere symptoms and do not require hospitalization. In hospitalized patients, it has been shown that about 15% of patients are treated in the ICU. Importantly, in a study from New York, 12% of hospitalized patients were reported to be placed on mechanical ventilation. In ventilated patients, the survival was found to be only 12% [16]. Despite the availability of several therapeutic agents being studied for COVID-19, all remain investigational, and supportive treatment is considered the therapeutic mainstay in affected patients.

Various infections have been known to be associated with an increased risk of thrombosis. There are several possible explanations of thrombosis in infections: (1) activation of platelets, (2) shift of the coagulation cascade towards procoagulant state, (3) stasis in the setting of prolonged immobilization, and (4) vascular endothelial dysfunction. The above-mentioned changes are consistent with Virchow’s triad, which is widely recognized as the pathophysiological foundation of venous thrombosis formation [17]. It is suggested that the inflammation-induced thrombosis from an infectious trigger has more procoagulant potential when compared with noninfectious inflammatory conditions. Viral models in animals of infections such as Ebola, influenza, and human immunodeficiency virus (HIV) have shown that viruses can induce the expression of tissue factor in phagocytes that in turn activate the extrinsic pathway of the coagulation cascade [18]. The activation of macrophages has been shown to induce deposition of fibrin in multiple organs [19]. In addition, in mice infected with a lethal dose of influenza virus leading to acute respiratory distress syndrome (ARDS), increased platelet aggregation, endothelial damage, and release of inflammatory cytokines manifesting as the presence of diffuse pulmonary microvascular thrombi have been observed [20].

In patients with COVID-19, it was estimated that up to a third of critically ill patients develop thromboembolic events [21]. Interestingly, life-threatening thrombosis has been noted to occur frequently in affected patients despite full-dose anticoagulation [22]. In all hospitalized patients with COVID-19, multiple laboratory tests are obtained initially. High fibrinogen and D-dimer can be indicative of procoagulant state. Fibrinogen (factor I) is a plasma protein involved in both primary and secondary hemostasis. Of note, there is some evidence that very high levels of fibrinogen can act as antithrombin and, therefore, compromise clot stability [23]. D-dimer is a protein fragment that is formed after the degradation of factor XIIIa cross-linked fibrin. It is cleared in the liver and spleen. Therefore, in patients with asplenia and liver failure, D-dimer can circulate in blood for a prolonged time [24]. Detection of normal levels of D-dimer is helpful in ruling out venous thromboembolism (VTE). In contrast, high levels of D-dimer are nonspecific and can be seen in patients with VTE as well as malignancy, sepsis, and other inflammatory conditions. It was reported that patients with very high levels of D-dimer (> 5000 µg/L) have high predictive value for serious disease, including VTE (40.1%), cancer (28.9%), sepsis (24.4%), and complicated trauma/surgery (24.4%) [25]. Imaging tests [bilateral compression ultrasonography of the legs and CT pulmonary angiography (CTPA)] are diagnostic tests of choice in patients with suspected VTE. In patients hospitalized with COVID-19, imaging tests such as CTPA may not always be readily available given healthcare strain due to the ongoing pandemic. Therefore, quantitative assessment of D-dimer is widely used to assess the risk of VTE and initiate empiric treatment with anticoagulation in high-risk patients. Of note, there are no universally accepted guidelines on D-dimer trends and the decision on anticoagulation in affected patients. Those decisions are mostly made based on physician discretion and hospital protocol.

In general, AAO is an extremely rare condition. Its true incidence is unknown, with numbers being obtained from case series. This is in contrast to arterial thrombosis leading to acute limb ischemia with an incidence rate of 1.5 per 100,000 person-years [26]. In 2019 the Swedish Nationwide Vascular Database reviewed patients with AAO from 1994 to 2014 and estimated an incidence rate of 3.8 per 1 million person-years, with the average individual being 70 years of age, presenting with acute limb ischemia with a history of cardiac disease, hypertension, and current smoking [1]. Le Berre *et al* reported a nonfatal case of acute aortic thrombosis in a 71-year-old male with COVID-19. A CT angiogram of the aorta revealed an accidental finding of a free-floating thrombus in the descending aorta. The patient was treated with therapeutic enoxaparin [6]. While our case likely highlights the first reported case of SARS-CoV-2-induced AAO, the association between viral infection and arterial thrombi has been previously identified [27, 28]. Unlike venous thromboembolic events proposed to be mostly related to hemostasis and coagulopathy, it has been postulated that arterial thrombus is driven more so by endovascular injury rather than stasis given the dynamic nature of the arterial wall [7].

Treatment options for acute arterial thrombosis include anticoagulation, thrombolysis, mechanical thromboembolectomy, and clot bypass. The choice of therapy depends on the timing and the setting of the artery involved, such as a coronary artery or a cerebral artery. Acute treatment with thrombolytics that cause the degradation of fibrin such as tPA is one such example where the timing of arterial thrombosis is crucial in its use [29]. Other common medical therapies include antiplatelet agents such as cyclooxygenase inhibitors, P2Y12 inhibitors, glycoprotein IIb/IIIa inhibitors, and protease-activated receptor 1 inhibitors [30]. Newer studies are beginning to investigate the utility of direct oral anticoagulants such as apixaban and rivaroxaban in peripheral arterial disease and other arterial thromboses [31, 32].

Acute aortic thrombosis, as compared with coronary, cerebral, and peripheral arterial thrombosis, is rare. There are no specific guidelines on management of acute aortic thrombosis as literature is mostly relegated to case reports.

In summary, the nascent COVID-19 pandemic has raised global concern regarding the role of viral infection and its effect on hypercoagulability. There is ongoing debate as to whether individuals should be actively monitored for severe thromboembolic events due to COVID-19 and if the initiation of therapeutic anticoagulation is warranted. Our case demonstrates that life-threatening hypercoagulability of large arterial vessels leading to occlusion can develop in patients with COVID-19, leading to hemodynamic collapse.

## Data Availability

The data that support the findings of this study are available from the corresponding author upon reasonable request.

## Abbreviations

**SARS-CoV-2**: Severe acute respiratory syndrome coronavirus 2
**SARS**: Severe acute respiratory syndrome
**COVID-19**: Coronavirus disease 2019
**AAO**: Acute aortic occlusion
**ED**: Emergency department
**CXR**: Chest X-ray
**BiPAP**: Bilevel positive airway pressure
**RT-PCR**: Reverse transcription polymerase chain reaction
**ICU**: Intensive care unit
**AAA**: Abdominal aortic aneurysm
**CMP**: Complete metabolic panel
**CBC**: Complete blood count
**DVT**: Deep vein thrombosis
**MAP**: Mean arterial pressure
**tPA**: Tissue plasminogen activator
**CT**: Computed tomography
**RNA**: Ribonucleic acid
**ACE2**: Angiotensin-converting enzyme-2
**HIV**: Human immunodeficiency virus
**ARDS**: Acute respiratory distress syndrome
**VTE**: Venous thromboembolism
**CTPA**: CT pulmonary angiography
**MERS**: Middle East respiratory syndrome
**WHO**: World Health Organization
